# Drones, automatic counting tools, and artificial neural networks in wildlife population censusing

**DOI:** 10.1002/ece3.8302

**Published:** 2021-11-03

**Authors:** Dominik Marchowski

**Affiliations:** ^1^ Ornithological Station, Museum and Institute of Zoology Polish Academy of Sciences Gdańsk Poland

**Keywords:** bird monitoring, birds’ reactions to drones, machine learning, microbiological tools in ecological research, unmanned aerial vehicle, waterbirds

## Abstract

1. The use of a drone to count the flock sizes of 33 species of waterbirds during the breeding and non‐breeding periods was investigated.
2. In 96% of 343 cases, drone counting was successful. 18.8% of non‐breeding birds and 3.6% of breeding birds exhibited adverse reactions: the former birds were flushed, whereas the latter attempted to attack the drone.
3. The automatic counting of birds was best done with ImageJ/Fiji microbiology software – the average counting rate was 100 birds in 64 s.
4. Machine learning using neural network algorithms proved to be an effective and quick way of counting birds – 100 birds in 7 s. However, the preparation of images and machine learning time is time‐consuming, so this method is recommended only for large data sets and large bird assemblages.
5. The responsible study of wildlife using a drone should only be carried out by persons experienced in the biology and behavior of the target animals.

## Graphical abstract

The experiment carried out on 33 species of waterbirds shows the effectiveness of the use of the drone in population censusing, 96% of 343 cases, drone counting was successful. The best automatic counting tool was microbiology software ImageJ/Fiji and Machine learning using neural network algorithms – DenoiSeg.

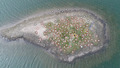

## 1 INTRODUCTION

Next to dealing with the consequences of climate change, biological diversity conservation has become one of the most important challenges for humanity (Díaz et al., 2006). The total loss of some species and the rapid decline of others have taken on a so far unknown dynamic (Hooper et al., 2012). Some species will probably not be described at all before they become extinct (Costello et al., 2013). Therefore, we should make every effort to use new technological solutions in ecological research in such a way that the knowledge gained thereby can be effectively used in nature conservation (Arts et al., 2015). In a rapidly changing world, we need ecological research methods that are fast, effective, and minimally invasive, so that we will be able to react dynamically to negative changes in the environment (Díaz‐Delago et al., 2017).

Large vertebrates, especially birds, have been regarded as indicators of the state of the environment (Amat & Green, 2010), and numerous long‐term bird monitoring programs have been set up in many places around the world (e.g. Farina et al., 2011; Niemi et al., 2016; Reif, 2013). New initiatives are constantly emerging, and because of this ever‐denser network of research programs, we are acquiring an increasingly precise model of ecological processes on Earth (Gregory & Strien, 2010). To meet this challenge, we need new, more effective methods and tools.

The use of Unmanned Aerial Vehicles (hereafter drones) in ecological research has already been described in research on breeding (e.g. Chabot et al., 2015; Corregidor‐Castro et al., 2021; Ratcliffe et al., 2015) and non‐breeding birds (e.g. Hodgson et al., 2018; Jarrett et al., 2020), as well as marine (e.g. Adame et al., 2017; Koski et al., 2015) and terrestrial mammals (Hu et al., 2020; Vermeulen et al., 2013). This has turned out to be an effective method for studying not only larger vertebrates, mainly birds and mammals, but also reptiles (Elsey & Trosclair, 2016), as well as for other ecological studies (Michez et al., 2016; Puttock et al., 2015). Nonetheless, research using drones is still at an early stage, and further studies are needed in order to establish both methodological standards and the actual effectiveness of working with this tool (Barnas et al., 2020). Apart from efficiency and time saving, an important issue is the invasiveness of this method and the safety of the studied object. Initial research in this area has already been carried out on a limited group of species (e.g. Jarrett et al., 2020; Vas et al., 2015). Traditional methods of counting waterbirds in breeding colonies involve human entry into the colony (Bibby et al., 2000), and the accurate counting of nests in such a situation requires extensive experience on the part of the observer and is prone to errors in estimation (Afán et al., 2018; Brisson‐Curadeau et al., 2017; Magness et al., 2019). An additional important factor is the disturbance of birds during a human visit to the colony, as this can cause birds from the entire colony or a large part of it to leave their nests, which may lead to brood losses (Fuller et al., 2018; Sardà‐Palomera et al., 2017).

Waterbirds often make use of hard‐to‐reach habitats, such as islands in water bodies or wetlands (Bibby et al., 2000; Valle & Scarton, 2019). Being bioindicators of environmental quality, they are also a frequent object of monitoring studies (Amano et al., 2017; Amat & Green, 2010). Therefore, the use of a drone for research on this group of animals should be doubly beneficial because (1) areas hard to reach on foot are easily and quickly accessed, and (2) disturbance of birds is limited, as there is no need to enter a breeding colony or disturb a flock of non‐breeding birds.

The next step in drone research is the analysis of photographs (Descamps et al., 2011; Hollings et al., 2018). Various approaches to this stage have been used, ranging from manual counting (Afán et al., 2018), through various types of semi‐automatic techniques, to advanced methods using artificial intelligence (Corregidor‐Castro et al., 2021; Descamps et al., 2011; Jarrett et al., 2020; Magness et al., 2019). As regards modern techniques of gathering ecological data, we are starting to face analytical issues (Shin & Choi, 2015) comparable to those in other fields, such as microbiology or biochemistry. The principle of similarities of natural structures, such as the similarities of a river network to a blood vessel network (e.g. LaBarbera & Rosso, 1989; Neagu & Bejan, 1999), may be applicable. Bird assemblages can resemble microbial assemblages, and the use of medical software to count birds has already been attempted (Pérez‐García, 2012). Since such software is commonly used for counting microorganisms, it has been tested many times and its precision confirmed (Barbedo, 2012). In the case of aerial photos, it seems justified to use analytical methods previously reserved for areas, such as microbiology, such as the ImageJ/Fiji open software platform used for automatic object counting (Schindelin et al., 2012) or the Passing Bablok regression, used to compare methods in clinical laboratory work (Bilić‐Zulle, 2011). Therefore, the present study utilized software dedicated to the study of microorganisms or tissues (ImageJ/Fiji) in order to compare its effectiveness with the manual method and other semi‐automatic bird counting techniques.

I thus focused on the effectiveness of a population censusing method using a drone, its invasiveness, the automated analysis of the data obtained by the drone, and the application of Artificial Intelligence (AI) for interpreting the results. The field study was carried out on colonial breeding waterbirds and gregarious waterbirds forming flocks during the non‐breeding period. I selected various species that occupy different habitats: these can be divided into four categories – open water, arable fields and meadows, wetlands, and islets.

My research questions were as follows: (1) Will it be possible/safe to count nests/incubating birds/individuals using a drone over a colony or a flock? (2) Will it be possible to identify similar species from a distance without endangering the birds? (3) Will the appearance of a drone over the breeding colony or flock cause the birds to react and, if so, in what way do they react? (4) Is automatic counting using dedicated software and Machine Learning applicable to bird censusing?

## 2 METHODS

### 2.1 Study area and species

The study was carried out on colonial and gregarious species of waterbirds in northern Poland (Europe). Most of the observations were made in the lower course and estuary of a large lowland river – the Lower Oder Valley (site‐center location in decimal degrees: Longitude – 14.413200, Latitude – 53.085000). The observations were made in areas known for their importance for waterbirds, during the breeding season, migration, and the wintering period. (Ławicki et al., 2010; Marchowski et al., 2018).

The study covered a total of 33 waterbird species, including 15 breeding and 28 non‐breeding species. The responses of 10 species to the drone in both the breeding and non‐breeding periods were compared.

### 2.2 Drone parameters

A DJI Phantom drone (version 4 Pro V2.0) was used for the fieldwork. This is a remotely controlled quadcopter device with a total weight of 1388 g, equipped with a camera capable of taking both still photographs (max quality: 5472 × 3648 pixels) and videos (max quality: 4096 × 2160 pixels), with the option of continuous tracking of unrecorded images transmitted to the display coupled with the remote control. Each photo records the geographic location and altitude in the metadata.

### 2.3 Species identification

To assess the possibility of accurate species identification using a drone, series of photos were taken at different heights from 5 to 100 m of gulls in a non‐breeding flock consisting of two species – Black‐headed Gull *Chroicocephalus ridibundus* and Herring Gull *Larus argentatus sensu lato*. An experienced ornithologist was then asked to identify the birds in the photos. The number of pixels each bird covered at each height was calculated. Generalized Linear Models from a binominal distribution were then analyzed to determine the influence of distance on species identification and the critical distance at which similar species could not be identified. The analysis was performed in the R environment (R Core Team, 2021).

### 2.4 Behavioral study

The distance from the observer to the surveyed sites was usually several hundred meters, but never less than 100 m. This precluded any influence on the part of the observer on the birds’ behavior. But if birds were scared away because of the observer's presence, no attempt was made to fly the drone. The flight height was set at about 100 m – the same as recommended for bird counts from aircraft (Meissner, 2011). From this height, the planned census area was scanned for birds. If a flock or individual birds were spotted on the remote controller display, a photo was taken, and the birds were approached. Up to a height of 30 m, birds were approached diagonally. If after reaching 30 m, when the birds were still not responding to the drone, and the species was not known or a flock contained birds of several similar‐looking species, the drone was moved over the birds in order to identify the species. The distance was shortened while maintaining the height, after which the drone's height was lowered, usually to about 15–20 m (less often to about 10 m, exceptionally even less; see Table S1 for the details). The birds’ reactions (if the terrain permitted them to be seen) were recorded by a second observer using a spotting scope mounted on a tripod. The birds’ reactions were also monitored in real time via the display on the drone's remote controller. The reaction of the birds was recorded throughout the drone's approach to the birds or the colony, so that it should have been possible to identify species‐specific reaction distances. The behavior of the birds was observed on a sample plot of approximately 40,000 m² (200 × 200 m), but if the site was smaller (e.g., a small lake, pool or islet), the entire area was treated as one sample plot.

Studies were carried out at 27 locations from 2017 to 2021, and 92 drone missions were flown, from 1 to 12 at each location. The locations were divided into five habitat types: 1 – open water (41 missions), 2 – wetland (28 missions), 3 – island (17 missions), 4 – farmland/meadow (18 missions), and 5) other (Grey Heron colony in a forest, two missions). During a single mission (drone departure and return), the birds were counted and their reaction to the drone was determined. From one to eight bird species were recorded during one drone flight. From 1 to 39 repeated drone missions took place per species, so as 33 species were recorded, a total of 343 separate records were obtained (Table 1, Table S1).

**TABLE 1:** List of species surveyed using a drone, broken down into breeding and non‐breeding birds, and the number of repeated drone missions for each species

No.	Species	Breeding	Non‐breeding	Number of repeated drone missions
1	Mute Swan *Cygnus olor*	Y	Y	25
2	Whooper Swan *Cygnus cygnus*	N	Y	13
3	Greater White‐fronted Goose *Anser albifrons*	N	Y	6
4	Bean Goose *Anser fabalis*/*serrirostris* ^a^	N	Y	20
5	Greylag Goose *Anser answer*	Y	Y	39
6	Mallard *Anas platyrhynchos*	Y	Y	39
7	Northern Pintail *Anas acuta*	N	Y	3
8	Eurasian Teal *Anas crecca*	N	Y	1
9	Eurasian Wigeon *Mareca penelope*	N	Y	3
10	Gadwall *Mareca strepera*	Y	Y	6
11	Pochard *Aythya ferina*	N	Y	10
12	Greater Scaup *Aythya marila*	N	Y	24
13	Tufted Duck *Aythya fuligula*	N	Y	22
14	Common Scoter *Melanitta nigra*	N	Y	5
15	Long‐tailed Duck *Clangula hyemalis*	N	Y	6
16	Common Goldeneye *Bucephala clangula*	N	Y	22
17	Goosander *Mergus merganser*	N	Y	6
18	Red‐breasted Merganser *Mergus serrator*	N	Y	6
19	Great Crested Grebe *Podiceps cristatus*	Y	Y	6
20	Great Cormorant *Phalacrocorax carbo*	N	Y	8
21	Grey Heron *Ardea cinerea*	Y	Y	7
22	Great Egret *Ardea alba*	N	Y	2
23	Eurasian Coot *Fulica atra*	N	Y	4
24	Common Crane *Grus grus*	Y	Y	15
25	Black‐headed Gull *Chroicocephalus ridibundus*	Y	Y	12
26	Common Gull *Larus canus*	Y	Y	8
27	Mediterranean Gull *Ichthyaetus melanocephalus*	Y	N	3
28	*Larus argentatus sensu lato* ^b^	Y	Y	7
29	Great Black‐backed Gull *Larus marinus*	N	Y	3
30	Little tern *Sternula albifrons*	Y	N	3
31	Common Tern *Sterna hirundo*	Y	N	12
32	Black Tern *Chlidonias niger*	Y	N	10
33	Whiskered Tern *Chlidonias hybrida*	Y	N	3

^a^ Bean Goose complex (two species: Tundra Bean Goose *Anser fabalis* and Taiga Bean Goose *Anser serrirostris*).

^b^ The group of closely related large gulls in the study area are Herring Gull *Larus argentatus* and Caspian Gull *Larus cachinnans*.

My main research question was whether it would be possible/safe to conduct bird research using a drone. How do birds react to a drone: do they ignore it, attack it, or does the drone flush them? The birds’ reactions to the drone were first divided into two basic categories: reaction and no reaction (code: no reaction = 0). The reaction category comprised the following subcategories – a bird: 1 – moved slowly away; 2 – dived; 3 – was flushed over a short distance but remained in the sample plot or quickly returned; 4 – was scared away, exhibited a panic reaction, left the sample plot, and did not return; 5 – attacked the drone. Whenever a bird was observed to react, the drone was immediately withdrawn.

Reactions 4 and 5 were classified as “unfavourable”, that is, situations where the study of birds using a drone would not be recommended. No reaction (0) and non‐invasive reactions (1–3) were placed in a separate category. Statistical significance was tested using the chi‐square goodness of fit. The statistical analyses were carried out using the software program R (R Core Team, 2021).

The second aim was to test the ability to count birds in non‐breeding aggregations or nests in breeding colonies using a drone. After initially ascertaining from a long distance whether the birds were responding to the drone's presence, attempts were made to approach the colony, with photos being taken continuously. If it was noticed that the drone had disturbed the birds in any way, it was recalled beyond the disturbing distance. The dates of drone flights over the breeding colonies were adjusted to take place when the colony was at the egg incubation stage. As a result, most of the nests in the colony, represented by an incubating bird, were visible.

### 2.5 Postprocessing – counting birds on photos

Photos from a Black‐headed Gull colony were used to assess the effectiveness of bird counting using various methods. In colonies of this species, Common Tern *Sterna hirundo* and other gull species – Herring Gull *Larus argentatus sensu lato*, Common Gull *Larus canus*, and Mediterranean Gull *Ichthyaetus melanocephalus –* also nested in smaller numbers. However, for the purposes of assessing the effectiveness of various counting methods, birds were not divided into species, so the basic unit in this assessment was the bird.

### 2.6 Manual and semi‐automatic counting

#### 2.6.1 Layer method

The primary method, regarded as a proxy method (see below for more details), uses Photoshop software to apply a layer to the counted object. This can be an unfilled ellipse or rectangle. The layer is then duplicated and moved to the next object. When a layer is duplicated, a sequential copy number is assigned to it so that the last layer number plus one (the first layer) gives the number of individuals in the count.

#### 2.6.2 Manual method

This involves printing an image of the colony or part of it and then marking off the counted birds or nests. The marked birds are grouped into tens; with larger colonies, supergroups multiplied by 10 are formed into sets of 100 individuals.

#### 2.6.3 Count tool method

Another approach is to use the object counting tool in Adobe Photoshop (Adobe Photoshop PS 2020: Image >Analysis >Count Tool). With this software, the clicks are counted automatically, with the consecutive number being displayed next to the clicked object.

### 2.7 Automatic counting

#### 2.7.1 Photoshop automatic count method

One method of automatic counting uses the Adobe Photoshop (PS 2020) software tool for the automatic counting of objects (Image >Analysis > Select Data Points >Custom > Object Counting). The image must be prepared so that the software knows what to count. Using image processing tools, such as *contrast*, *brightness*, *exposure*, and *threshold*, the counted objects need to be exposed against the background. The best results are achieved when the objects to be counted can be seen against a completely uniform background. Then, using the Wand tool, the background is selected, and the selection inverted. The next step is to open the Measurement Log and record the measurements. The software will automatically count the selected objects.

#### 2.7.2 ImageJ/Fiji method

Another automatic counting method is the one used for counting microorganisms or cell structures – the Analyze Particles tool in the open‐source Java image processing software – ImageJ (Grishagin, 2015; Schroeder et al., 2020) and its extended version of Fiji (Schindelin et al., 2012). This method requires image processing each time before automatic counting is applied. Tools such as *contrast*, *brightness*, *exposure*, *threshold*, etc. should be used in such a way that the counted objects are clearly visible against the background.

#### 2.7.3 Machine learning + ImageJ/Fiji method

**FIGURE 1:**
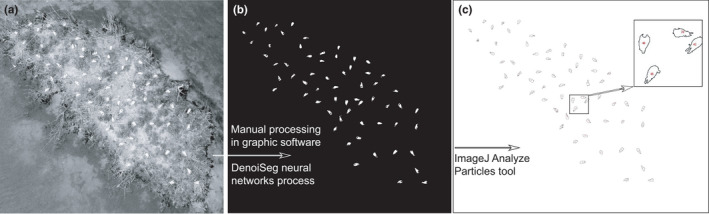
Bird counting process using two approaches: ImageJ/Fiji and Machine learning + ImageJ/Fiji. In the first method, the step from image (a) to (b) is performed manually, whereas in DenoiSeg it is performed automatically using the neural network model. The step from picture (b) to (c) in both methods is the same

This is a similar method to the above as it uses the Analyze Particle tool in the ImageJ/Fiji platform at the final stage. The manual stage of processing the image is skipped and a neural network‐based algorithm is used to display the objects in the background. This method was applied using the ImageJ/Fiji platform, the DenoiSeg tool from the CSBDeep plug‐in – the neural network algorithm for instance segmentation (Buchholz et al., 2020). The machine learning process requires an appropriate graphics card and the installation of several drivers and software that operate in the background of the main software (CUDA Toolkit, GPU support, TensorFlow, cuDNN SDK, Phyton etc.; see https://imagej.net/plugins/denoiseg and Buchholz et al., 2020 for more details). Having set up the computer, I followed these three steps. (1) Creating manual segmentations for six of the images – labelling (requiring several hours of manual work). (2) Training the neural network – this was based on 70 noisy images and six labelled ones. I created two folders for training and two for validation (X_train, Y_train, X_val, and Y_val), which were then uploaded to the DenoiSeg software (the technical details are described step by step in Buchholz et al., 2020 and are available at: https://imagej.net/plugins/denoiseg). The network training process took about 36 h (computer RAM 8 GB; processor: Intel (R) Core (TM) i5‐7300HQ CPU @ 2.50GHz; graphics adapter: NVIDIA GeForce GTX 1060). The result of the process was a model of the trained neural network. (3) Prediction: I used the trained model to segment the image. The separation of objects from the background usually took a second or two. The image prepared in this way could then be further processed using the Analyze Particles tool in ImageJ/Fiji (Baviskar, 2011). Figure 1 highlights the difference between the ImageJ/Fiji only and Machine Learning +ImageJ/Fiji approaches.

### 2.8 Comparison of counting methods

Forty‐three photos of bird assemblages from the Black‐headed Gull breeding colony were selected at random and analyzed using different methods. The photos were mostly of Black‐headed Gulls, but a few other species (Common Tern and other gull species) were also present though much less frequently. In this analysis, they were all counted together. Species with a different (darker) coloration, such as Greylag Goose, were eliminated during the preparation of the photo for counting and not counted.

**FIGURE 2:**
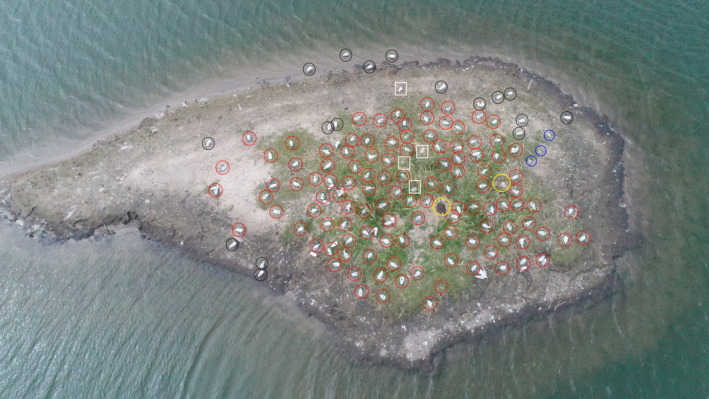
Results of the Layer method: this yields a precise count of the birds incubating in a breeding colony, as well as identification of the species and their activities, that is, whether a bird is incubating or standing on the ground. Red circles – incubating Black‐headed Gulls, black circles – Black‐headed Gulls standing on the ground or in the water, yellow circles – incubating Greylag Geese and nests with eggs, blue circles – incubating Common Terns, white squares – birds performing an unidentified activity, or possibly dead

A stopwatch was used to measure the time needed to process and count the birds. The Photoshop Layer method allows one to zoom in on the image to check whether the bird should be added to the result. This yielded much more detail than was needed for this study (bird). The Layer method enables the identification of species in a group and the activity being performed by a bird (e.g., in a breeding colony, whether it is incubating, Figure 2). Given the precision of the Layer method, the results obtained with it were taken as reference values (proxy method). Then, the count precision was compared with the proxy method using different methods. All the other methods used in this study (Manual, Click Tool, Photoshop automatic count tool, ImageJ/Fiji and DenoiSeg + ImageJ/Fiji) were compared to the Layer method separately using Passing Bablok Regression (Bablok and Passing, 1985; see also below for more details).

Passing Bablok Regression is a linear regression procedure with no special assumptions regarding sample distribution or measurement errors. The result does not depend on the assignment of the methods or instruments. The regression equation (*y *= *a *+ *bx*) revealed constant (the regression line's intercept (a)) and proportional (the regression line's slope (b)) differences with confidence intervals of 95% (95% CI). The confidence intervals explain whether their value differs from zero (0) for the intercept and one (1) for the slope only by chance. Thus, if 95% CI for the intercept includes the value zero it can be concluded that there is no significant difference between the obtained intercept value and zero, and that there is no constant difference between the two methods. Correspondingly, if 95% CI for the slope includes the value one, it can be concluded that there is no significant difference between the obtained slope value and one, and that there is no proportional difference between the two methods. In such a case, we can assume that x = y and that there is no significant difference between the methods, so both can be used interchangeably (Bablok and Passing, 1985, Bilić‐Zulle, 2011). The minimum sample size required to assess the effectiveness of the method is 40 (Bilić‐Zulle, 2011), so in my comparison, I used a sample size of 43 randomly selected images. The statistical analyses were carried out using the software program R (R Core Team, 2021).

## 3 RESULTS

### 3.1 Species identification

**FIGURE 3:**
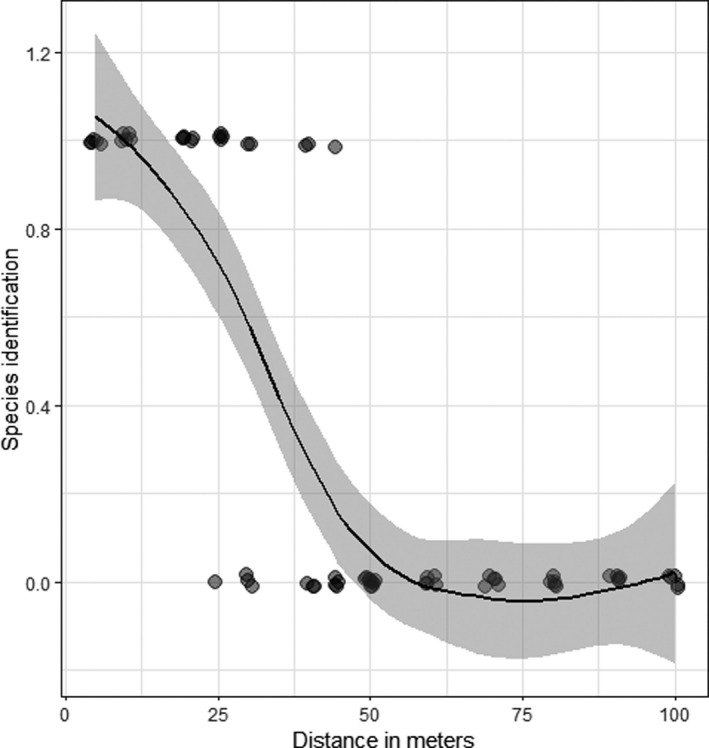
The influence of distance on the identification of species in aerial photos, as exemplified by Black‐headed Gull (*Chroicocephalus ridibundus*) and Herring Gull (*Larus argentatus sensu lato*)

The greater the distance, the harder it is to identify a species (Table 2, Figure 3), so the distance coefficient is significant (*p* = .0001, Table 2). A species could be identified correctly up to a height of about 25 m; above this height, it was usually possible to identify only the subfamily (Larinae), and Black‐headed Gulls could not be distinguished from Herring Gulls.

**TABLE 2:** Results of a Generalized Linear Model showing the effect of distance on species identification as exemplified by Black‐headed Gull (*Chroicocephalus ridibundus*) and Herring Gull (*Larus argentatus*)

	Estimate	*SE*	*z* value	*p* value
(Intercept)	6.11967	1.68486	3.632	.000281***
Distance	−0.18368	0.04749	−3.868	.00011***

Note

Asterisks indicate significance codes: 0 '***', .001 '**', .01 '*', .05 '.', .1 ' '.

One hundred percent of the birds in an image with more than 10,000 pixels could be identified to species level. This figure dropped to 81% if the resolution of the images was from 1000 to 3500 pixels, to 40% at resolutions of 300–1000 pixels, to 5% for 100–300 pixels, and 0% if there were fewer than 100 pixels.

### 3.2 Reaction to the drone

The ratio of drone response to no response was almost half and half (52.8% – reaction, 47.2% – no reaction, chi‐square test: *χ* ^2^ = 1.052; *p* = .30, *n* = 343).

However, the drone‐induced response was usually insignificant (birds moved slowly away or flew away for a short distance): most drone missions (84.8%, *n* = 52) yielded a combination of no response and insignificant reactions. Significant reactions, that is, birds being flushed from the sample plot or attacking the drone, together accounted for 15.2% (chi‐square test: *χ* ^2^ = 10.828; *p* < .001). This indicates that negligible (not significant for birds) reactions or no reaction constitutes the statistically significant majority (Table 3). The feasibility of counting birds using a drone was confirmed in 331 cases (*n* = 343, Table 3).

**TABLE 3:** Results of chi‐square goodness‐of‐fit tests regarding the reaction of birds to the appearance of a drone

Variable	*n*		%		The result of the goodness of fit test ‐ chi‐square
	0	1	0	1	
Drone reaction all species	0	343	0	100	–
Reaction NOT/YES	162	181	47.2	52.8	χ^2^ = 1.053; *p *= .30
NAR reaction NOT/YES	291	52	85.4	15.2	χ^2^ = 10.83; *p* < .001***
Count possible	0	343	0	100	–
NOT/YES	12	331	3.5	96.5	χ^2^ = 15.14; *p* < .001***

Note

NAR, non‐acceptable response.

Asterisks indicate significance codes: 0 '***', .001 '**', .01 '*', .05 '.', .1 ' '.

The average distance from the drone to the birds when an unacceptable reaction occurred was 35.8 m (± min.–max. 15 m–50 m). In the case of breeding birds, an unacceptable reaction took place in three cases (*n* = 83) and involved only one species – Black Tern *Chlidonias niger* – the reaction each time being an attempted attack on the drone. Where non‐breeding birds were concerned, unacceptable reactions occurred in 49 cases (*n* = 260), all of them involving the birds flying off beyond the sample plot. None of the non‐breeding birds attempted to attack the drone. The average flock size among birds displaying unacceptable reactions was 317 individuals and was slightly higher than that of birds exhibiting no or negligible reactions (291) (Table 4).

**TABLE 4:** Mean distance of the drone from birds

No.	Species	Mean distance	Mean dist. of reaction #0	Mean dist. of reaction #1	Mean dist. of reaction #2	Mean dist. of reaction #3	Mean dist. of reaction #4	Mean dist. of reaction #5
1	*Cygnus olor*	13	13	NA	NA	NA	NA	NA
2	*Cygnus cygnus*	18	12	8	NA	28	43	NA
3	*Anser albifrons*	44	35	NA	NA	NA	49	NA
4	*Anser fabalis*/*serrirostris* ^a^	32	23	30	NA	NA	41	NA
5	*Anser anser*	26	15	13	NA	29	44	NA
6	*Anas platyrhynchos*	16	11	13	NA	30	43	NA
7	*Anas acuta*	15	NA	15	NA	NA	NA	NA
8	*Anas crecca*	15	NA	NA	NA	15	NA	NA
9	*Mareca penelope*	15	NA	15	NA	NA	NA	NA
10	*Mareca strepera*	10	11	10	NA	NA	NA	NA
11	*Aythya ferina*	31	35	26	NA	NA	NA	NA
12	*Aythya marila*	23	32	19	24	NA	20	NA
13	*Aythya fuligula*	25	30	20	25	NA	29	NA
14	*Melanitta nigra*	31	35	25	NA	NA	NA	NA
15	*Clangula hyemalis*	30	35	25	NA	NA	NA	NA
16	*Bucephala clangula*	24	33	24	23	20	21	NA
17	*Mergus merganser*	20	25	18	18	NA	NA	NA
18	*Mergus serrator*	35	NA	35	NA	NA	NA	NA
19	*Podiceps cristatus*	14	14	NA	NA	NA	NA	NA
20	*Phalacrocorax carbo*	14	11	20	NA	13	NA	NA
21	*Ardea cinerea*	29	32	NA	NA	26	NA	NA
22	*Ardea alba*	25	NA	NA	NA	25	NA	NA
23	*Fulica atra*	14	10	30	NA	NA	NA	NA
24	*Grus grus*	25	10	20	NA	25	50	NA
25	*Chroicocephalus ridibundus*	15	12	NA	NA	40	NA	NA
26	*Larus canus*	13	13	NA	NA	NA	NA	NA
27	*Ichthyaetus melanocephalus*	15	15	NA	NA	NA	NA	NA
28	*Larus argentatus sensu lato* ^b^	9	9	10	NA	10	NA	NA
29	*Larus marinus*	15	13	20	NA	NA	NA	NA
30	*Sternula albifrons*	15	15	NA	NA	NA	NA	NA
31	*Sterna hirundo*	13	13	15	NA	10	NA	NA
32	*Chlidonias niger*	24	15	NA	NA	25	NA	37
33	*Chlidonias hybrida*	9	9	NA	NA	NA	NA	NA

Note

The mean distances of the initial reaction to the drone approach and type of reaction. Type of reaction – a bird: #1 – moved slowly away; #2 – dived; #3 – was flushed over a short distance but remained in the sample plot, or quickly returned; #4 – was scared away, exhibited a panic reaction, left the sample plot, and did not return; #5 – attacked the drone; #0 – displayed no reaction; NA – no reaction of any type was recorded.

^a^ Bean Goose complex (two species Tundra and Taiga Bean Goose *Anser fabalis*/*serrirostris*).

^b^ The group of closely related large gulls in the study area is Herring Gull *Larus argentatus* and Caspian Gull *Larus cachinnans*.

Adverse (unacceptable) reactions were recorded in 13 species (*n* = 33). *Anser* geese were the most sensitive to the drone's presence with 35.8% of unacceptable reactions. Gulls were the least sensitive to the drone's presence: no unacceptable reactions were observed, and the birds were completely indifferent to the drone in 88.8% of cases (Table 5).

**TABLE 5:** The behavior of birds in response to the drone, broken down into groups of similar species

Group of species	No of obs.	%: code #0	%: code #1	%: code #2	%: code #3	%: code #4	%: code #5
*Anser*	65	41.5	9.2	0	13.8	**35.8**	**0**
*Anas* group^a^	24	41.6	37.5	0	12.5	**8.3**	**0**
Diving birds^b^	119	28.6	37.0	14.3	3.4	**16.8**	**0**
Gulls	36	88.8	5.6	0	5.6	**0**	**0**
Terns	28	75.0	3.8	0	10.7	**0**	**10.7**

Note

Reaction codes – a bird: #0 – displayed no reaction; #1 – moved slowly away; #2 – dived; #3 – was flushed over a short distance but remained in the sample plot, or quickly returned; #4 – was scared away, exhibited a panic reaction, left the sample plot, and did not return; #5 – attacked the drone.

Bold values are adverse (unacceptable) reactions # 4 and # 5.

^a^ Ducks from the Anatini tribe consisted of the following genera: *Anas*, *Mareca*, and *Spatula*.

^b^ Group consisting of diving ducks from the genera *Aythya*, *Bucephala*, *Melanitta*, *Mergus* and *Clangula*, Coot *Fulica atra*, Great Crested Grebe *Podiceps cristatus*, and Cormorant *Phalacrocorax carbo*.

### 3.3 Comparison of counting methods

**FIGURE 4:**
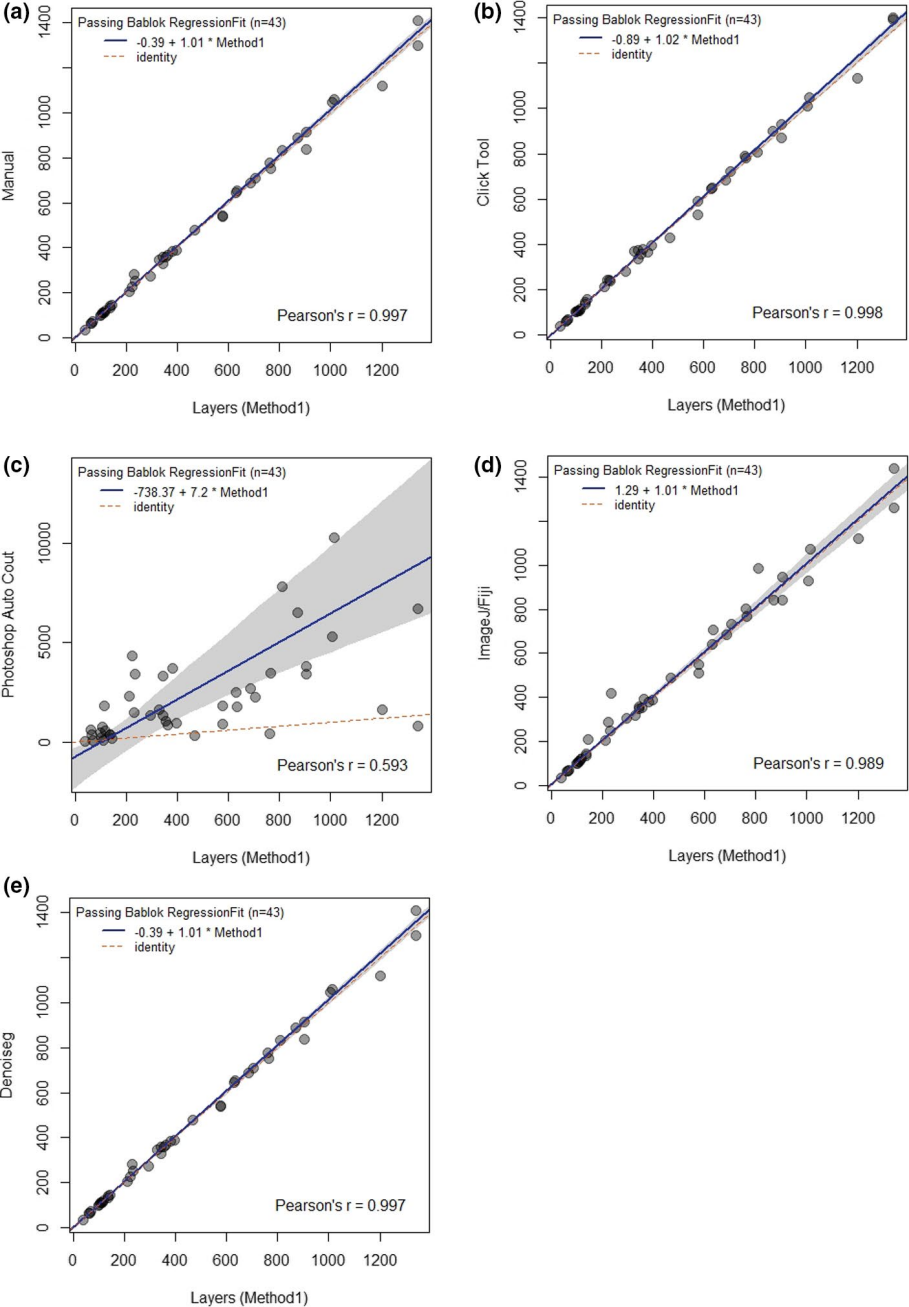
Comparison of methods using the Passing Bablok regression. The graphs show the records with the regression line (solid line), the confidence interval for the regression line (dashed lines), and the identity line (*x *= *y*, dotted line). Comparison between the proxy methods and other methods: (a) – Manual, (b) – Click Tool, (c) – Photoshop Autocount, (d) – ImageJ/Fiji, and (e) – DenoiSeg + ImageJ/Fiji

For all methods except one – the Photoshop automatic count – the 95% CI of the difference from zero includes zero. Hence, there is no significant difference between the intercept value and the zero value, and there is no consistent difference between these methods. Likewise, if the 95% CI (of the difference from one) for the slope includes the value 1, there cannot be a significant difference between the slope and unity, and there is no proportional difference between the methods. As with the intercept, all the methods meet this assumption, except the Photoshop automatic count (Table 6, Figure 4). The above calculations prove that the results of the counting methods – Manual, ImageJ/Fiji, Click Tool and DenoiSeg – do not differ significantly from the Layer proxy method and can therefore be used interchangeably. The only method that is not recommended is Photoshop Autocount, as the results obtained with it differ significantly from the proxy method.

**TABLE 6:** Results of Passing Bablok regression models, comparison of counting methods

Method comparison	Estimate	L 95%CI	U 95%CI
Layer vs. Manual			
Intercept	−0.8876245	−3.8653823	3.355620
Slope	1.0227596	0.9968978	1.034173
Layer vs. ImageJ/Fiji			
Intercept	1.289855	−4.7397276	12.120016
Slope	1.007246	0.9576697	1.055058
Layer vs. Photoshop Autocount			
Intercept	−738.36782	−2272.77634	−293.20543
Slope	7.198686	4.940249	10.80274
Layer vs. Click Tool			
Intercept	−1.756221	−5.413764	0.9931358
Slope	1.027441	1.000000	1.0416667
Layer vs. Denoiseg + ImageJ/Fiji			
Intercept	−0.385159	−5.0712107	3.044472
Slope	1.014134	0.9858247	1.030146

The Layer method was the most time‐consuming, the average time needed to count 100 birds being 893 s. Birds were counted the fastest using the neural networks machine learning method (DenoiSeg) + ImageJ/Fiji – an average of 100 birds in 7 s. All the methods except DenoiSeg exhibited a significant correlation between the number of birds in the group and the time needed to count them: the more birds, the longer it took to count them. With the DenoiSeg method, the number of individuals in a group did not affect the time needed to count them (Table 7).

**TABLE 7:** Average time (in seconds) needed to count 100 individuals in a group of birds and correlation with the size of the group

Method	Average time to count 100 individuals	Pearson's reg. Time/number of ind.	Significance
Layer	927 (*n *= 43)	0.969	*p *< .001***
Manual	223 (*n *= 43)	0.933	*p *< .001***
Click Tool	172 (*n *= 43)	0.891	*p *< .001***
ImageJ/Fiji	64 (*n *= 43)	0.562	*p *< .001***
DenoiSeg + ImageJ/Fiji	7 (*n *= 43)	0.282	*p *= .064

Note

Asterisks indicate significance codes: 0 '***', .001 '**', .01 '*', .05 '.', .1 ' '.

## 4 DISCUSSION

### 4.1 Field study

The drone proved to be a useful tool for the safe study of colonial and gregarious waterbirds: their flocks could be counted in more than 96% of cases. It also turned out to be minimally invasive: out of 343 birds/missions, no dangerous event was recorded, such as a collision of a bird with the drone or a permanent nest abandonment because of a bird being scared away. A similar conclusion was reached during research conducted in Australia based on 97 flight hours (Lyons et al., 2018) and in Wales (UK, Rush et al., 2018). However, studies of birds nesting on cliffs in Canada (Common Guillemot *Uria aalge* and Thick‐billed Guillemot *Uria lomvia*) showed that in several cases, drones scared birds off their nests, which were then plundered by predatory birds (Brisson‐Curadeau et al., 2017). The strongest reactions to the presence of the drone were displayed by *Anser* geese in the non‐breeding period, chiefly large flocks foraging on farmland. Similar results were obtained in Scotland, where, in addition, a dependence on flock size was demonstrated: the larger the flock, the greater the chance of its responding to a drone (Jarrett et al., 2020). My results indicate that the group of birds reacting adversely to the drone was slightly larger than that exhibiting a moderate reaction or none at all. Birds in the non‐breeding period reacted more strongly to the drone's appearance – 18.8% of adverse reactions. Breeding birds, on the other hand, appeared to be indifferent to the drone, undesirable behavior being manifested in only 3 (3.6%) cases out of 83. Chabot et al. (2015) drew similar conclusions from their study in a Common Tern colony. In our case, however, these undesirable behaviors (observed in Black Tern) were manifested in attempts to attack the drone. This is potentially more dangerous than when the birds are scared away over a long distance, as a bird–drone collision may ensue; drone attack behavior has been reported in Australia (Lyons et al., 2018). One brief attempt at attacking a drone was also recorded during the above‐mentioned study in Wales (Rush et al., 2018).

Despite the promising results of this study, the effectiveness and minimal invasiveness of the drone, the use of a drone for performing bird counts should be approached with great caution. Species‐specific responses should be considered. This is because, as my research (Table 4) shows, but also a study conducted in Canada (Brisson‐Curadeau et al., 2017), some species may react more strongly than others, which may lead to dangerous situations, such as collisions with a drone or a predator plundering a nest. The persons conducting the research must have a good knowledge of the study area, so that in the event of an emergency, the drone can be landed quickly. They must also be experienced in bird biology and behavior in order to be able to predict and prevent dangerous situations by withdrawing the drone at the right moment (Rush et al., 2018).

At this point, it is important to separate the impacts of a drone on breeding and non‐breeding birds. In the first case, great care should be taken not to disturb the birds at all, as there is a risk of the nest being abandoned or of possible losses in the breeding colony caused by many birds being scared off and the ensuing confusion (Brisson‐Curadeau et al., 2017; Chabot et al., 2015). However, it should be emphasized that breeding birds quickly get used to the drone (Chabot et al., 2015; Rush et al., 2018). Hence, assuming there are no predators near the colony that can quickly rob a nest (Brisson‐Curadeau et al., 2017), a brief absence from it in response to a drone does not endanger the birds (Rush et al., 2018). In the case of non‐breeding birds, one should aim for a situation where the birds completely ignore the drone, because a drone being flown over large aggregations of feeding or roosting waterbirds could elicit energetically costly flight responses, increased stress, and effective loss of available habitat (Jarrett et al., 2020). In the case of breeding birds, attention should also be paid to the fact that an incubating bird may not show signs of fear but may feel it. Currently, we need research to address the question of whether a minimal behavioral response of breeding birds, or none at all, coincides with the same body response at a physiological level.

The reactions of birds to an approaching drone are not easy to predict (Brisson‐Curadeau et al., 2017). Much probably depends on their individual characteristics (Herrmann, 2016). In a flock, the impulse to flee would presumably be a reaction of the most skittish individuals. Very skittish individuals are more likely to be present in larger flocks, which is probably why such flocks react more quickly to the appearance of a drone and from a greater distance than smaller ones (Jarrett et al., 2020). Hence, when flying a drone toward birds of the same species, their reactions may sometimes differ in similar circumstances. In non‐breeding geese, for example, strong reactions, such as flying off over a long distance, were recorded when the drone was still quite a long distance (over 40 m) away from them, whereas they did not react at all when the drone was only 24 m away (see also Table 4 for more examples). This demonstrates that the rule according to which the farther away the drone, the less the reaction it causes is not always applicable. An aspect not explored in this article is distinguishing between the effect on birds of the appearance of a drone (visual reaction) and that of the sound it makes. For example, we can use a special type of propeller to reduce the sound emitted by the drone and to test the reaction of birds or other animals.

In the ideal case, the birds completely ignore the drone. If a flock of birds consists of one species or when we simply want to determine the overall number of birds without separating them into species, the drone can be flown relatively high up, at 50 or even 100 m above the ground (Figure 3), depending on the contrast of the birds’ coloration with the background (Corregidor‐Castro et al., 2021). However, in a situation where a flock of birds consists of two or more species that resemble each other closely (e.g., different species of gulls and terns), the drone has to be brought down much nearer to them. In the present study, I tested the possibility of distinguishing two different gull species, easily identified in the field – Black‐headed Gull and Herring Gull *sensu lato*. Problems with identification from the drone started above a height of 25 m (~1500 px; Figure 3). In the case of species extremely similar in appearance, like Black‐headed Gull and Mediterranean Gull, the distance of the drone from the bird would have to be less than 25 m (~1500 px) for positive identification. In identifying some species or determining other parameters, such as reading rings/tags, photos from above may not be sufficient and photography from the side should be considered. This poses some risk, especially among breeding birds that are incubating eggs (Brisson‐Curadeau et al., 2017; Corregidor‐Castro et al., 2021). However, it never happened during this study that a drone flying over a gull breeding colony toward one particular individual caused other individuals to fly off in a panic, as happened in the study by Chabot et al. (2015). The birds remained on their nests; even the one that was the target of the drone did not usually leave the nest. This occurred only exceptionally (see Table S1), and then just one bird would leave the nest, returning immediately as soon as the drone was withdrawn. Comparison of the method of counting birds from a drone (even in exceptional cases when the drone is flown as close as 10 m) with the traditional method of counting nests in a colony, that is, human entry and counting nests (Bibby et al., 2000), shows that the drone method is much less invasive, and the results are far more precise (Brisson‐Curadeau et al., 2017; Corregidor‐Castro et al., 2021; Hodgson et al., 2018). At this point, it is also necessary to mention the dependence of distance on the quality of the photos taken with the drone's camera. This research shows the capabilities of a camera with a resolution of 5472 × 3648 pixels. Of course, with a better resolution or using a camera with optical zoom, the same effects can be obtained at a greater distance from the birds. When assessing the possibility of species identification and other parameters, such as behavior, egg count, or brood productivity, it is preferable to use the pixel count in the pictures rather than the distance from the object, as shown in this paper (see Results – species identification).

This research has shown that counting with a drone is not entirely non‐invasive, and that extreme caution should be exercised at all times. A single visit of a drone to a colony will probably not adversely impact its breeding success (Chabot et al., 2015). By contrast, a drone flying over a breeding colony or a flock of non‐breeding birds, often at low altitude, can have a negative effect on individual breeding success, elicit energetically costly flight responses, cause increased stress and the effective loss of available habitat (Jarrett et al., 2020; Sardà‐Palomera et al., 2017). Therefore, the frequent flying of large numbers of drones over flocks of birds for recreational purposes should be proscribed. Drones are becoming more and more affordable (Kyrkou et al., 2019), and the temptation for people to “play” with birds will have a decidedly negative impact on the latter.

The present study was conducted by a person with 30 years of experience in field ornithology, so his ability to predict bird behavior will have helped to avoid dangerous situations. At the present stage, of course, one can speculate that it is highly probable that people with less experience will pose a greater risk of dangerous situations for animals and humans. Hence, the use of drones in wildlife research should be legally regulated: a license should be issued for such work, and the persons involved should have passed an examination in animal biology and behavior.

### 4.2 Automatic counting

Bird counts using medic software were described by Pérez‐García (2012), who used the UHTSCSA Image Tool 3.0 software to perform a census of starlings. This software does not work everywhere, however; for example, it cannot be installed in the Windows 10 operating system. Other free software dedicated to birds was described by Descamps et al. (2011), who used it to count flamingos in breeding colonies. But this latter software has turned out to be quite difficult to use: one serious difficulty is that the basic language is French and that no other language versions are available. In contrast, while DotCount v1.2 is easy to use, a disadvantage is the closed source, so it cannot be used to create plugins or updates to improve its functionality; moreover, the latest version of this software stems from as long ago as 2012. In the future, the use of neural networks and machine learning in wildlife studies will undoubtedly become more important (e.g. Tabak et al., 2019; Villa et al., 2017). Promising results have been obtained with the use of CountEm software: typical relative standard errors after counting ~200 properly sampled animals in about 5 min are in the 510% range (González‐Villa & Cruz, 2019). Clearly, there are many different possibilities and solutions for automatic bird counting, but at present, ImageJ/Fiji (Grishagin, 2015) seems to be one of the best choices. As it is an open‐source platform, several programmers and biologists can work together to create new plugins. Some of them already use neural network‐based algorithms (Buchholz et al., 2020).

The automatic counting methods using the ImageJ/Fiji platform proved to be the best in this study, as they were the fastest and maintained the precision of the results (Tables 6 and 7, Figure 4). For small and medium bird concentrations, I recommend using the Analyse Particles tool in the ImageJ/Fiji software program. This does require pre‐treatment of each image each time, but with some practice, this can be done quite efficiently and the results will be precise.

In my study, I used photos from a Black‐headed Gull breeding colony, in which other species, such as Common Tern and other gull species (Mediterranean, Herring and Common), also nested, though not as frequently. All these species were counted together for this study. However, there is a possibility (not tested in this study) that automatic counting can cope with different species if they are essentially of different shapes and/or sizes. In the automatic counting tools (ImageJ/Fiji), this can be done by defining the pixel range and circularity of the objects to be counted (Grishagin, 2015). Thus, one can simply count all the similar species together or set the count to size and circularity corresponding to a particular species or group of species.

In the case of solutions using neural network algorithms, a one‐off count takes a very short time (100 birds in 7 s on average, Table 4), but to achieve this state, a lot of prior preparation is necessary, and the images require some preliminary assumptions. An undoubted advantage of the DenoiSeg approach is that advanced artificial intelligence technologies are easily applied (Buchholz et al., 2020). In other cases, the user has to possess coding skills in environments, such as Phyton or Java (e.g. Frank et al., 2010; Pedregosa et al., 2011). The computer on which the machine learning will take place has to have the appropriate hardware. Then, the images must be scaled in such a way that the objects are roughly the same size, and the learning time of the neural network can take up to several dozen hours. This method is therefore recommended in situations where a lot of data (images) have been acquired from long‐term monitoring programs, in large breeding colonies and in non‐breeding concentrations. When planning multi‐year monitoring, the learned models can then be used for producing images in subsequent years of monitoring.

## 5 CONCLUSIONS

The drone turned out to be a convenient tool that can be successfully used to count waterbirds during both the breeding and non‐breeding seasons. The methods presented in the article can be applied not only to waterbirds, but also to other groups of birds, not to mention other groups of vertebrates, such as antelopes, bison, and seals. To identify similar species, the drone needs to approach within a short distance from the bird; for instance, to distinguish Black‐headed Gulls from Herring Gulls this will be about 25 m, assuming a camera resolution of 5472 × 3648 pixels. For more closely similar species, the distance may have to be shorter, though longer distances are possible with better camera resolutions. Birds do not normally react to the drone, but this does not mean that it is always safe to fly a drone over them. Occasionally, birds have been observed to attack a drone, a situation that can lead to dangerous collisions. On the other hand, if a drone does force an incubating bird to leave the nest, this may be plundered in the meantime by predators. Drone surveys of birds should therefore be planned in such a way that the device is ignored by the birds. The automatic counting of birds using ImageJ/Fiji software, and more extensive trials and planned long‐term monitoring using DenoiSeg +ImageJ/Fiji turned out to be easy to use, precise, and fast.

## CONFLICT OF INTEREST

None declared.

## AUTHOR CONTRIBUTION

**Dominik Marchowski:** Conceptualization (equal); Data curation (equal); Formal analysis (equal); Funding acquisition (equal); Investigation (equal); Methodology (equal); Project administration (equal).

## ETHICAL APPROVAL

Permissions to carry out the study. Personal exemption from the prohibitions applicable to protected animal species pursuant to Art. 56 sec. 2 points 1 and 2 of the Act of April 16, 2004, on the protection of nature (Journal of Laws of 2013, item 627, as amended), scaring birds for scientific purposes of all protected species of birds in Poland was issued for on the basis of the decision of the General Director for Environmental Protection (issued annually, the last permit DZP‐WG.6401.102.2020.tł). The permit for game species was issued separately by the Minister of the Environment (DL‐III.6713.11.2018.ABR). The drone missions were performed on the basis of the relevant licenses and permits, operator number ‐ POL55b3419341c0h, valid until 22 March 2023, and A1, A2, and A3 licenses, pilot number – POL‐RP‐dd90349b468r, valid until 2 April 2026.

## Supporting information

Table S1

## Data Availability

The raw data on the basis of which the analyses were carried out are available at the Dryad Digital Repository: https://doi.org/10.5061/dryad.j6q573nfp.
